# Autologous bone graft in the treatment of post-traumatic bone defects: a systematic review and meta-analysis

**DOI:** 10.1186/s12891-016-1312-4

**Published:** 2016-11-09

**Authors:** Matheus Lemos Azi, Alessandro Aprato, Irene Santi, Mauricio Kfuri Junior, Alessandro Masse, Alexander Joeris

**Affiliations:** 1 Manoel Victorino Hospital, Conselheiro Almeida Couto square S/N, 40050-410 Salvador, Bahia Brazil; 2 Department of Orthopaedic Surgery, San Luigi Hospital of Orbassano, University of Turin, Regione Gonzole n.10, 10043 Turin, Italy; 3 AO Clinical Investigation and Documentation, Stettbachstrasse 6, 8600 Dübendorf, Switzerland; 4 Department of Biomechanics, Medicine and Rehabilitation of the Locomotor Apparatus - Ribeirão Preto Medical School, University of São Paulo (FMRP-USP), Av. Bandeirantes 3900, 14048-900 Ribeirão Preto, São Paulo, Brazil; 5 Department of Orthopaedic Surgery, University of Missouri, 1100 Virginia Avenue, Columbia, Missouri USA

**Keywords:** Bone graft, Segmental bone defect, Large bone defects, Bone reconstruction

## Abstract

**Background:**

This meta-analysis aimed to determine the bone union rate of bone defects treated with the different autologous bone graft techniques.

**Methods:**

The PubMed and the Cochrane Library databases were searched using the terms: ‘fracture’ AND (‘bone loss’ OR ‘defect’ OR ‘defects’) AND ‘bone graft’, restricted to English language, to human species, and to a publication period from January 1999 to November 2014. Data were extracted by one of the reviewers and then checked by the second. A quality of evidence score and a methodology score were used. Heterogeneity was assessed. A random effects model approach was used to combine estimates.

**Results:**

Out of 376 selected studies only 34 met the inclusion criteria. The summary pooled union rate was 91 % (95 % CI: 87–95 %) while union rate after additional procedures raised to 98 % (95 % CI 96–99 %). No association between union rate and bone defect size was found. (Univariable regression model: vascularized: *P* = 0.677; non-vascularized: 0.202. Multivariable regression model: vascularized: *P* = 0.381; non-vascularized: *P* = 0.226). Vascularized graft was associated with a lower risk of infection after surgery when compared to non-vascularized graft (95 % CI 0.03 to 0.23, *p* < 0.001).

**Conclusion:**

The results of this meta-analysis demonstrate the effectiveness of autologous graft for bone defects. Furthermore, from the available clinical evidence bone defect size does not seem to have an impact on bone union when treated with autologous bone graft techniques.

**Electronic supplementary material:**

The online version of this article (doi:10.1186/s12891-016-1312-4) contains supplementary material, which is available to authorized users.

## Background

Conventional autologous bone graft has become the most widely used treatment for bone defects over time. Several factors contributed to its widespread application: it is easy to obtain, it combines osteogenic, osteoinductive and osteoconductive properties, it does not raise immune response or transmit infectious diseases [1, 2]. Furthermore, autologous bone graft can be harvested in a variety of forms and sizes from different donor sites [1, 2].

Selection of the autologous graft type in the treatment of bone defects has been mostly based on defect size: several authors do not recommend the use of the non-vascularized graft in defects larger than 5 cm [1–3]. The more technical demanding vascularized bone graft method is considered the best choice for larger size defects [2, 3]. However, in recent years, advances in graft harvesting technique [4] and in wound environment recovery using the polymethylmethacrylate (PMMA) induced membrane technique [5], renewed the interest in the use of the non-vascularized autologous bone graft. Infection also plays a role in graft selection and a 2-stage approach with delayed grafting is sometimes necessary [6].

The primary objective of this meta-analysis is to determine the bone union rate of post-traumatic bone defects treated with the different autologous bone graft techniques. The secondary objective is to determine the rate of infection after this treatment.

## Methods

### Data collection and extraction

Prior to doing the electronic search, a written protocol was established according to guidelines for systematic reviews (AMSTAR, MOOSE and PRISMA) [7–9]. An electronic search was conducted in Medline restricted to English language, to human species, and to a publication period from January 1999 to November 2014. The search terms and Boolean operators used were: ‘fracture’ AND (‘bone loss’ OR ‘defect’ OR ‘defects’) AND ‘bone graft’. Additionally an electronic search was done in the Cochrane Library with the terms: fracture AND bone loss AND defect OR defects AND bone graft.

Two reviewers (MA, AA) independently scrutinized the list of titles of all the retrieved citations and, if necessary, the abstracts to determine usefulness of the article. The final selection was based on the full text version of the potentially relevant articles that were assessed independently by the reviewers. All references cited in these elected studies were manually searched along with the “related articles” researches in PubMed engine for additional relevant studies. Papers published by the same research group and studying the same factors were checked for duplicate data. Where duplication occured the less detailed paper was discarded.

We included only original reports that presented the results of at least ten cases of bone defects secondary to open fractures, post-traumatic nonunion or infected bone resection. The exclusion criteria were: bone defects after tumor resection; bone defects after reduction and fixation of closed metaphyseal fracture of long bones; studies with more than 25 % of the defect not located in long bones (forearm, humerus, femur or tibia); studies with more than 25 % of the defects treated with osteoconductive biomaterials in addition to the bone graft; cases with the use of osteoinductive factors in the graft; studies mostly about bone defects in children and studies that did not report the information about healing after treatment. When the information of each patient in a study was presented in the text and/or tables, cases that met the exclusion criteria were removed and the remaining patients were enrolled in the analysis.

Included studies were classified according the Oxford Centre for Evidence-Based Medicine system and a modified version of the Coleman methodology score [10] (Additional file 1). Data was extracted by one of the reviewers and then checked by the second. Disagreements were solved via discussion and consensus between the two reviewers. The following definitions were used for data extraction: primary union described as bone union achieved after bone grafting, secondary union as bone union achieved with a further surgery after the bone graft. Of note, a graft fracture was considered a union related complication only when the original study classified it in this manner, and cases with union before lost of follow up were considered as treated. Treatment failures were viewed as the loss of the graft in the postoperative period that required debridement and a new graft, the absence of bone union during follow-up or a new bone defect treatment (bone transport, amputation, etc.). Preoperative infection refers to the presence of infection (active or quiescent) or absence of it when bone defect treatment was implemented. Postoperative infection was infection reported as a complication after bone graft procedure. We considered that PMMA was used as an adjuvant in bone defect treatment (induced membrane technique) only when authors reported its use for this purpose.

### Assessment of publication bias

Susceptibility of the systematic review to publication bias was formally assessed with the Egger test [11].

### Quantitative data synthesis

To stabilize variance, the bone union proportions were subject to a Freeman-Tukey arcsine square root transformation and back-transformed according to Miller after quantitative data synthesis [12, 13]. With the normalized data, heterogeneity was assessed using both Cochran’s Q test and the inconsistency measure I^2^ suggested by Higgins [14]. A cut-off of *P* < 0.10 was used to indicate heterogeneity. Values of I^2^ equal to 25 %, 50 % and 75 % denoted a low, moderate and high degree of statistical heterogeneity. As data from a series of studies that had been performed independently are thought to be not functionally equivalent, a random effects model approach was used to combine estimates. Confidence intervals within studies were achieved using the exact binomial method. To perform a sub-group analysis, the studies were divided according to graft vascularization in two major categories: non-vascularized bone graft or vascularized bone graft. Analyses were performed using STATA (version 13.0) and Comprehensive Meta-analysis (version 2.0).

## Results

### Selection of studies

**Fig. 1:**
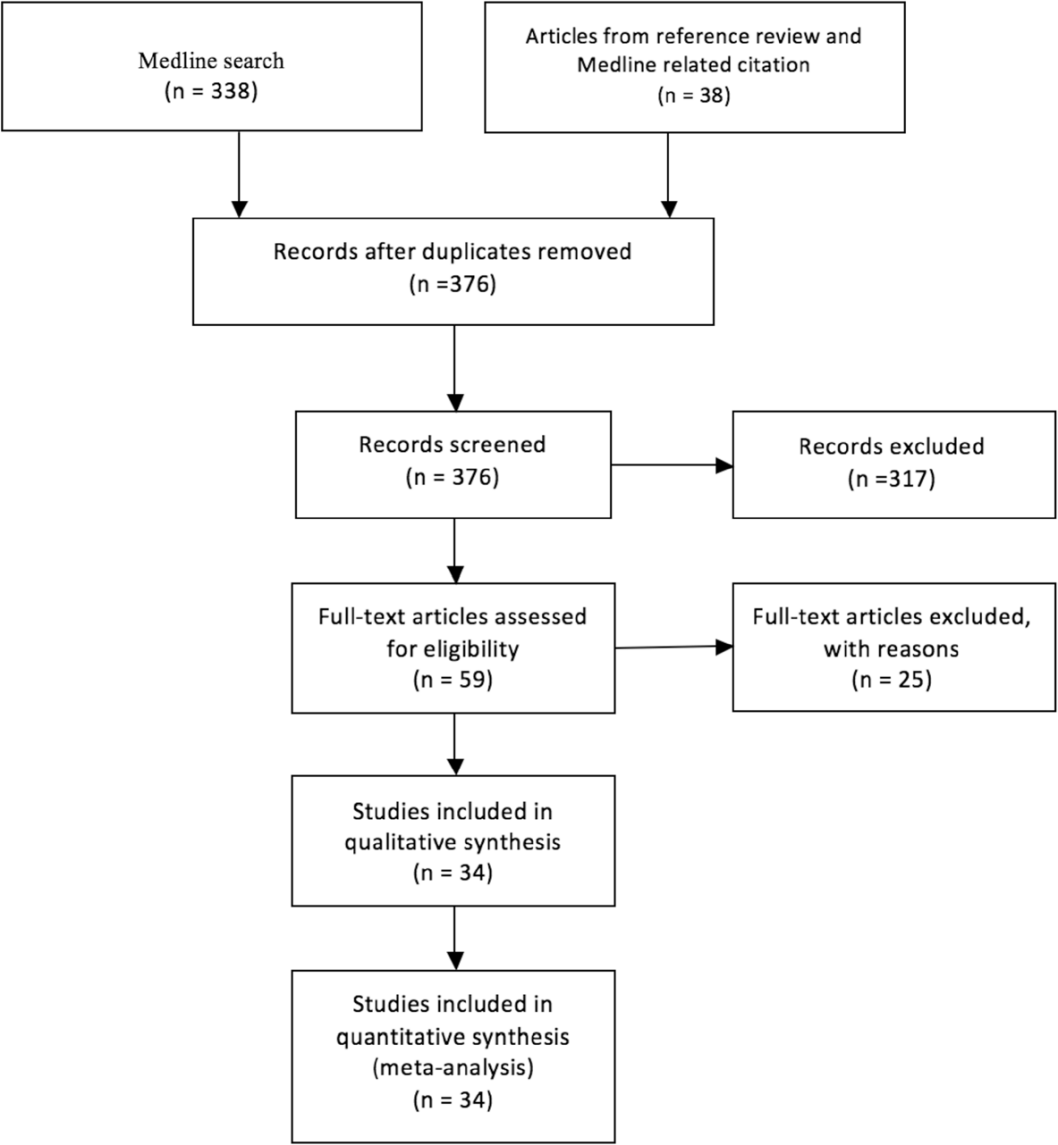
Flowchart of literature review

The Medline search resulted in 338 citations and after the abstract review 21 were considered as potentially eligible and all of them had the full version reviewed. References of these articles were manually screened and also the related citations tool resulting in further 38 potentially eligible articles, totaling 59 papers to review. The Cochrane Library search did not result in additional studies. After the full version review, 34 studies met the inclusion/exclusion criteria (Fig. 1) (Additional file 2). In seven of the 34 studies some cases were excluded from the analysis (Additional file 3). A total of 749 patients with 750 bone defects were included in this meta-analysis.

Concerning study characteristics, one was a randomized controlled trial, one was a prospective case series, three were retrospective comparative case series and 29 were retrospective case series (Table 1). Only Pelissier et al. [15] was a comparison between vascularized and non vascularized bone graft. The studies achieved 37.1 points (21 to 72) out of 100 in the quality assessment tool. The inter rater agreement in regards to the quality assessment between the reviewers was considerably high (ICC = 0.78; 95 % CI 0.75 to 0.94).

**Table 1:** Studies and cases descriptive characteristics

Authors	Year	Treatment period	Study type	LE	(n)	Age (y) [range]	Male/female	Bone defect location (femur/tibia/humerus/foream/other bones)	FU (mts)
Ring et al. [16]	2000	nr	R-CS	IV	15	48 [22 - 80]	9/6	-/-/15/-/-	31
Tu et al. [17]	2001	1990–1993	R-CS	IV	48	48 [15 - 62]	40/8	10/32/2/4/-	72
Toh et al. [18]	2001	1983–1998	R-CS	IV	19	53 [21 - 84]	17/2	-/19/-/-/-	98
Heitmann et al. [19]	2002	nr	R-CS	IV	12	43 [16 - 79]	7/5	-/-/12/-/-	tn
Muramatsu et al. [20]	2003	1985–2000	R-CS	IV	13	51 [27 - 80]	6/7	-/-/13/-/-	tn
Pelissier et al. [15]	2003	1984–1999	R(C)-CS	IV	40	tn	tn	tn	tn
Yajima et al. [21]	2004	1976–2000	R-CS	IV	20	37 [17 - 73]	16/4	9/8/-/2/1	64
Lee et al. [22]	2004	1982–2001	R-CS	IV	51	41 [15 - 66]	48/3	-/51/-/-/-	nr
Adani et al. [23]	2004	1993–2000	R-CS	IV	11	38 [16 - 65]	5/6	-/-/-/11/-	tn
Ring et al. [24]	2004	1983–2001	R-CS	IV	35	40 [21 - 66]	18/17	-/-/-/35/-	43
Yazar et al. [25]	2004	1993–2000	R-CS	IV	61	37.5 [10 - 82]	42/19	7/49/-/-/6	58
Safoury [26]	2005	nr	R-CS	IV	18	34 [22 - 46]	16/2	-/-/-/18/-	36
Jones et al. [27]	2006	2000–2003	RCT	II	15	38 [18 - 71]	13/2	-/15/-/-/-	tn
El-Sayed et al. [28]	2007	nr	R-CS	IV	12	25 [12 - 40]	11/1	-/8/2/2/-	24
Ristiniemi et al. [29]	2007	2000–2004	R-CS	IV	23	35 [14 - 75]	16/7	-/23/-/-/-	nr
Adani et al. [30]	2008	1994–2004	R-CS	IV	13	37 [21 - 62]	10/3	-/-/13/-/-	nr
El-Gammal et al. [48]	2008	1995–2004	R(C)-CS	IV	13	31.5 [nr]	11/2	-/13/-/-/-	38
Ryzewicz et al. [31]	2009	1998–2007	R(C)-CS	IV	18	34.2 [18 - 51]	11/7	-/18/-/-/-	nr
Allende et al. [32]	2009	1996–2008	R-CS	IV	10	32.8 [11 - 56]	9/1	-/-/4/6/-	tn
Cavadas et al. [33]	2010	2000–2008	R-CS	IV	41	nr [17 - 64]	39/2	-/41/-/-/-	nr
McCall et al. [34]	2010	2003–2007	P-CS	IV	21	30.6 [nr]	13/8	5/15/-/1/-	nr
Sun et al. [35]	2010	2005–2007	R-CS	IV	10	31 [16 - 50]	9/1	3/7/-/-/-	26
Apard et al. [36]	2010	nr	R-CS	IV	12	40.6 [18 - 74]	10/2	-/12/-/-/-	39
Zhen et al. [37]	2010	2000–2007	R-CS	IV	28	31.5 [17 - 56]	21/7	-/28/-/-/-	36
Chai et al. [38]	2010	2005–2007	R-CS	IV	16	31 [16 - 50]	10/6	-/9/-/4/3	18
Georgescu et al. [39]	2011	1997–2007	R-CS	IV	44	30.5 [5 - 66]	33/11	3/22/5/3/11	23
Chung et al. [40]	2011	1989–2007	R-CS	IV	10	25.3 [16 - 43]	8/2	-/10/-/-/-	41
Niu et al. [41]	2011	2003–2008	R-CS	IV	19	38.9 [18 - 61]	12/7	8/11/-/-/-	nr
Liang et al. [42]	2012	1996–2006	R-CS	IV	16	33.3 [21 - 46]	nr	16/-/-/-/-	83
Gulan et al. [43]	2012	1991–1998	R-CS	IV	10	30 [22 - 51]	10/0	-/10/-/-/-	144
Liang et al. [44]	2012	2001–2007	R-CS	IV	14	34.3 [23 - 48]	11/3	14/-/-/-/-	67
Gao et al. [45]	2012	2004–2006	R-CS	IV	18	34 [16 - 56]	13/5	7/11/-/-/-	40
Niu et al. [46]	2012	1993–2008	R-CS	IV	22	33.8 [17 – 60]	14/8	-/-/22/-/-	39
Özaksar et al. [47]	2012	1993–2009	R-CS	IV	21	32 [16 - 47]	19/2	-/21/-/-/-	74

*LE* level of evidence, *n* number of patients included in this review, *y* years, *mts* months, *FU* follow-up, *R* retrospective, *P* prospective, *CS* case series, *C* controlled, *RCT* randomized controlled trial, *nr* not reported, *tn* technical note (see Additional file 3)

### Publication bias

**Fig. 2:**
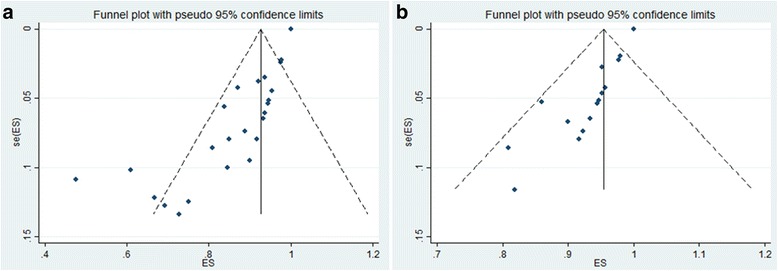
Funnel plot comparing proportion versus the standard error of proportion for the outcome of (**a**) primary union and (**b**) secondary union. The assessment of the Egger test was coupled with an informal visual inspection of the funnel plot where circles represent studies included in the meta-analysis. The solid vertical line indicates no union in terms of proportion. The outer dashed line indicate the triangular region within which 95 % of studies are expected to lie in absence of both bias and heterogeneity (random effect pooled proportion ± 1.96 × standard error of pooled proportion). Asymmetry about the pooled proportion line is consistent with the presence of publication bias

The shape of the funnel plot revealed evidence of asymmetry for both primary and secondary union (Fig. 2). The Egger’s test showed evidence of publication bias (*p* < 0.001 for primary union and *p* < 0.001 for secondary union).

### Bone union rate

Primary bone union was documented in 33 studies [15–47] (Q = 87.53, df = 32, *P* <0.001; I^2^ = 63.4 %) and secondary bone union was documented in 34 studies [15–48] (Q = 38.65, df = 33, *P* =0.23; I^2^ = 14.6 %). Union rates as primary ranged between 48 % and 100 % across eligible studies. Using random-effects weights, the summary (pooled) union rate was 91 % (95 % CI: 87–95 %). Union rates as secondary ranged between 81 % and 100 % across eligible studies. Using random-effects weights, the summary (pooled) union rate was 98 % (95 % CI 96–99 %).

For comparison of vascularized versus non-vascularized graft the study from Toh et al. [18] and the study from Muramatsu et al. [20] were omitted as they used a mixed technique. The study of Pelissier et al. [15] included patients treated either with vascularized or non-vascularized graft, thus this publication contributed to both groups in the sub-group analysis (Table 2).

**Table 2:** Graft type and healing rate per treatment

Authors	*n*	BD Mean (cm)	Treatment type	Donor area/associated technique (n cases per treatment)	Primary union	Secondary union
			Nonvascularized bone graft			
Ring et al. [16]	15	3 [2–6]	ICBG	ICBG (15)	93 %	93 %
Pelissier et al. [15]	16	4.3 [nr]	ICBG	ICBG (16)	75 %	81 %
Jones et al. [27]	15	4 [2.5–7]	ICBG	ICBG (15)	67 %	93 %
Ryzewicz et al. [31]	18	3.8 [2–6]	ICBG	ICBG (18)	89 %	94 %
Niu et al. [46]	22	tn [tn]	ICBG	ICBG (22)	95 %	100 %
Gulan et al. [43]	10	4 [2–7]	ICBG	ICBG (10)	100 %	100 %
Ring et al. [24]	35	2.2 [1–6]	Multiple DS	ICBG (33)/Ulna (4)	100 %	100 %
El-Sayed et al. [28]	12	7 [6–10]	Multiple DS	ICBG (8)/Fibula (12)	92 %	92 %
Niu et al. [41]	19	nr [nr]	Multiple DS	ICBG (19)/Fibula (2)	95 %	95 %
Ristiniemi et al. [29]	23	5.2 [3.5–10]	ICBG + biomembrane	ICBG (23)/biomembrane (23)	61 %	96 %
Allende et al. [32]	10	3.2 [1–7]	ICBG + biomembrane	ICBG (10)/biomembrane (10)	100 %	100 %
Apard et al. [36]	12	8.7 [6–15]	ICBG + biomembrane	ICBG (12)/TCF (4)/biomembrane (12)	92 %	92 %
McCall et al. [34]	21	6.6 [2–14.5]	RIA + biomembrane	RIA (21)/biomembrane (18)	48 %	81 %
			Vascularized bone graft			
Heitmann et al. [19]	12	9.2 [8–12]	Free one DS	Fibula (12)	75 %	92 %
Lee et al. [22]	51	10.5 [4.5–17]	Free one DS	Fibula (51)	92 %	98 %
Adani et al. [23]	11	8.7 [6–13]	Free one DS	Fibula (11)	73 %	82 %
Safoury [26]	18	nr [nr]	Free one DS	Fibula (18)	94 %	100 %
Adani et al. [30]	13	10.5 [6–16]	Free one DS	Fibula (13)	69 %	92 %
El-Gammal et al. [48]	13	12.6 [nr]	Free one DS	Fibula (13)	nr	100 %
Sun et al. [35]	10	9.5 [6–17]	Free one DS	Fibula (10)	90 %	100 %
Zhen et al. [37]	28	nr [9–17]	Free one DS	Fibula (28)	100 %	100 %
Chai et al. [38]	16	13.8 [5–20]	Free one DS	Fibula (16)	100 %	100 %
Liang et al. [44]	14	6.9 [5–9]	Free one DS	Fibula (14)	100 %	100 %
Liang et al. [42]	16	16.4 [14–20]	Free one DS	Fibula (16)	94 %	100 %
Gao et al. [45]	18	9.2 [7–14]	Free one DS	Fibula (18)	100 %	100 %
Özaksar et al. [47]	21	10 [6–18]	Free one DS	Fibula (21)	81 %	95 %
Toh et al. [18]	19	4.9 [1–11]	Pedicle one DS	Fibula (19)	95 %	100 %
Chung et al. [40]	10	5.4 [4–8]	Pedicle one DS	Fibula (10)	100 %	100 %
Yajima et al. [21]	20	9.6 [3–24]	Free + pedicle one DS	Free fibula (16)/pedicle fibula (4)	85 %	90 %
Georgescu et al. [39]	44	8.2 [4–14]	Free one DS	Free rib (44)	98 %	98 %
Pelissier et al. [15]	24	9.8 [nr]	Free multiple DS	Fibula (12)/iliac (10)/arm (2)	88 %	88 %
Yazar et al. [25]	62	11.7 [6–18]	Free multiple DS	Fibula (50)/iliac (6)/rib (6)	87 %	95 %
Cavadas et al. [33]	41	nr [4–17]	Free multiple DS + biomembrane	Fibula (38)/iliac (3)/biomembrane (32)	98 %	100 %
			Nonvascularized and vascularized bone graft			
Muramatsu et al. [20]	13	1.8 [1–4]	Free multiple DS + ICBG	Fibula (8)/femur (4)/scapula (1)/ICBG (8)	85 %	100 %
Tu et al. [17]	48	10.2 [6.5–19]	Free multiple DS + ICBG	Fibular (41)/iliac (4)/Rib (3)/ICBG (48)	94 %	100 %

*n* number of bone defects, *ICBG* iliac crest bone graft, *DS* graft donor site, *TCF* tricalcium phosphate, *tn* technical note (see Additional file 3)

**Fig. 3:**
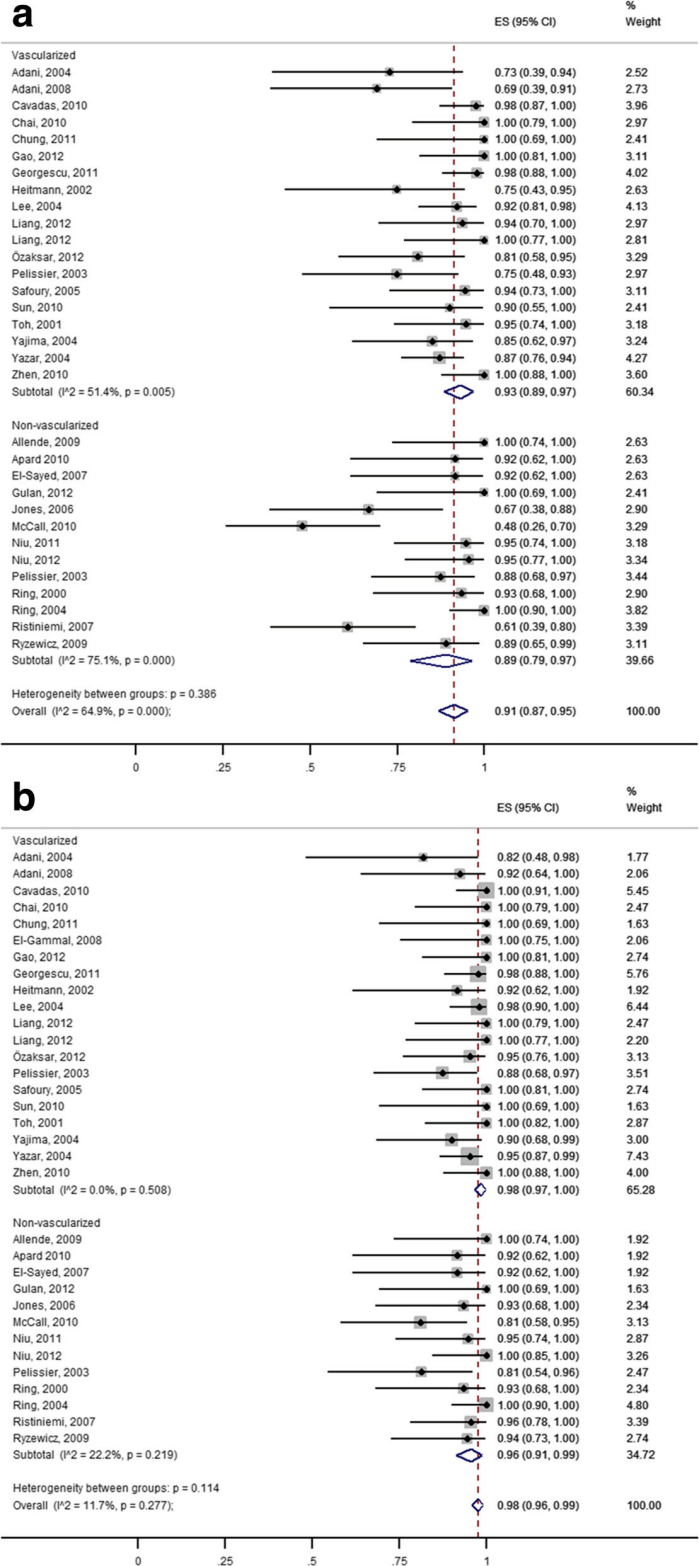
Forest plot of bone union (% of union rates) in patients with vascularized and non-vascularized bone graft (random effects model). **a** Primary union **b** Secondary union

When analyzing the primary bone union, significant intra-group heterogeneity was observed (Vascularized: Q = 37.07, df = 18, *P* < 0.01; I^2^ = 51.4 %; Non-vascularized: Q = 47.48, df = 12, *P* < 0.001; I2 = 74.7 %). However, there was no statistical difference between the two groups (*P* = 0.372) supporting the pooling of all studies into one pooled measure. Using random-effects weights, the summary (pooled) union rate was 93 % (95 % CI: 89–97 %) for the vascularized group and 89 % (95 % CI: 79–97 %) for the non-vascularized group (Fig. 3a).

Meta-regression was performed to investigate potential sources of heterogeneity within study for primary bone union. The main factor investigated was bone defect size additionally adjusted for age and proportion of female patients. Both univariable and multivariable meta-regression did not show any association of union rate and bone defect size (Univariable: vascularized: *P* = 0.677; non-vascularized: 0.202. Multivariable: vascularized: *P* = 0.381; non-vascularized: *P* = 0.226).

When analyzing the secondary bone union, no significant intra-group heterogeneity was observed (Vascularized: Q = 18.22, df = 19, *P* = 0.508; I^2^ = 0.0 %; Non-vascularized: Q = 15.20, df = 12, *P* = 0.231; I^2^ = 21.0 %), neither difference between groups was noted (*P* = 0.106). Using random-effects weights, the summary (pooled) secondary union rate was 98 % (95 % CI: 97–100 %) for the vascularized group and 96 % (95 % CI: 91–99 %) for the non-vascularized group (Fig. 3b).

### Infection pre and post-treatment

**Fig. 4:**
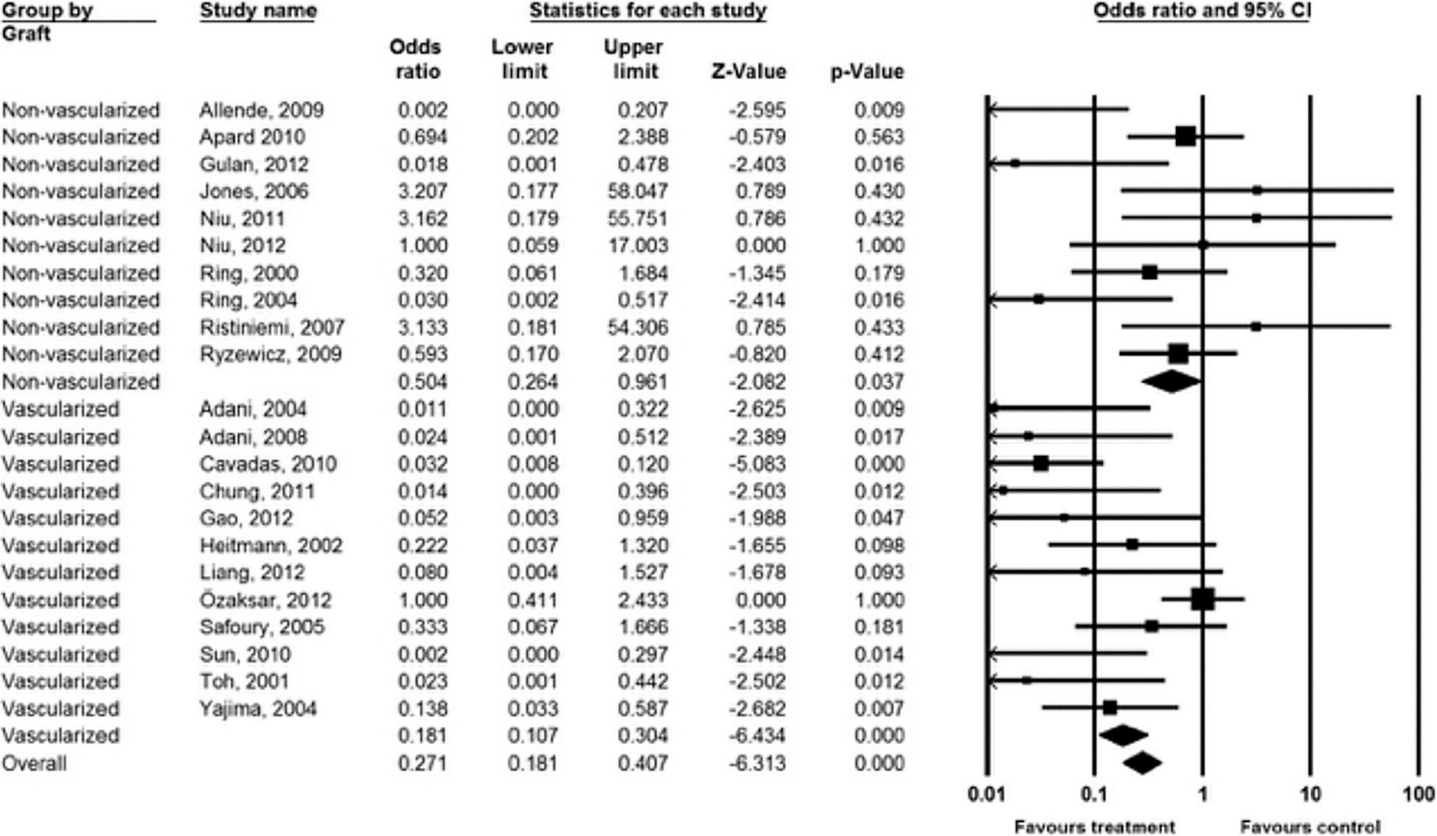
Forest plot of the odds ratio of bone infection before and after bone graft treatment stratified by type of graft (vascularized and non-vascularized)

Infection status of the cases was reported pre- and post-operative in 22 studies [16, 18, 19, 21, 23, 24, 26, 27, 29–33, 35, 36, 40, 41, 43–47]. The pooled estimate of mean effect size showed about 6-fold decrease of infection after treatment compared with pre-operative situation (OR = 0.17 (95 % CI 0.08 to 0.36), *p* < 0.001; Q = 58.6, *p* < 0.001, df = 21, I^2^ = 64.2 %). Therefore, a subgroup analysis was performed. A significant decrease of post-treatment infection was observed among the vascularized graft group (*n* = 12; OR = 0.08 (95 % CI 0.03 to 0.23), *p* < 0.001) but not in the non-vascularized group (*n* = 10; OR = 0.43 (95 % CI 0.15 to 1.22), *p* = 0.114). Moreover, a statistical difference between the two groups was found (Q = 4.350; *P* = 0.037) (Fig. 4).

As heterogeneity in the subgroup analysis may be due to the presence of outlying studies, a sensitivity analysis was conducted excluding the studies that presented the highest OR (Jones et al. [27], Ristiniemi et al. [29] and Niu et al. [41]). After these studies were excluded a moderate degree of heterogeneity (I^2^ = 63.7 %) was found. In the sensitivity analysis there was no statistical difference between the two techniques (Q = 1.146; *P* = 0.284) and the non-vascularized group also showed a statistically significant decrease of post-operative infection (*n* = 7; OR = 0.207 (95 % CI 0.06–0.77)).

Additionally, investigation of heterogeneity was performed by means of meta-regression including age, percentage of females and months of delay from injury to treatment. No variables showed a significant association with the risk of post-treatment infection.

## Discussion

### Bone union

The primary bone union rate expected for the bone graft techniques is 91 %. In some circumstances, additional procedures such as the change of a broken implant, compression in the nonunion site or cancellous graft in nonunion areas at bone ends, may be necessary and they raised the union rate to 98 % in published studies (Additional file 4) [15, 17–23, 25–31, 33–35, 39, 41, 42, 46–48].

### Defect size as a guide to select graft

Only few studies presented a description of the method used to define and measure the bone defect [27, 29, 31, 46]. Small defects that might have been susceptible to spontaneous regeneration were present in some studies. They were treated not only with non-vascularized graft but also with vascularized bone graft.

Studies about vascularized bone grafts have been performed on larger bone defects but association of union rate and bone defect size wasn’t found between the vascularized and non-vascularized grafts. Limitations of this conclusion include also a potential selection bias: some recent studies about non-vascularized graft were excluded because of the addition of growth factor or biomaterial to the graft. Despite the limitation of this study, our data suggests that selection of graft technique shall not be guided only by defect size. Patient expectations, surgeon experience, soft tissue condition and a trained staff to perform microsurgery are elements that must be carefully judged before making a decision on the graft to be used.

### Infection pre- post-treatment

The pooled estimate of mean effect size showed a decrease of infection after treatment compared with the pre-operative situation. However, these findings should be interpreted with caution due to the presence of a moderate degree of statistical heterogeneity. According to the results of this meta-analysis, vascularized graft showed a significant decrease of post-treatment infection. Again, this conclusion is limited. Infection definition varies between the included studies and several different surgical techniques were used. Although we cannot give evidence to support this recommendation, most of the studies suggest a two step reconstruction as the standard approach to manage infected bone defects: an extensive debridement, followed by antibiotic treatment before graft surgery [16, 21, 26, 29, 31, 32, 36, 40, 43, 44]. Furthermore, some of the studies use PMMA as a local antibiotic delivery and/or due to its ability to induce a biological membrane at the defect site [21, 29, 32–34, 36].

### Overall completeness and applicability of evidence

The included studies provide the most complete information available concerning union rates after autologous graft for bone defects; however, different factors may have added to the heterogeneity of the pooled results, such as different treatment techniques, different sample sizes reflecting different levels of experience, incomplete information about complications. Additionally, information regarding surgical steps was limited in several studies. Finally, data concerning potential confounding factors, such as patients selection criteria, soft tissue treatment and definition of complications were also incomplete.

### Quality of the evidence

The overall quality of the included studies is poor. Most of them are nonrandomized observational studies with serious limitations. There was evidence of publication bias for primary and secondary bone union, with higher union rates in bigger studies. Overall sample size allows obtaining several statistically significant results. However, the level of evidence of these findings is low or very low due to the heterogeneity of the pooled data and the risk of bias caused by the studies’ design.

## Conclusion

This study states the effectiveness of autologous graft for bone defects. Overall union rate was 91 % while union rate after additional procedures raised to 98 % in published studies. Available clinical evidence does not show a direct relation between bone defect size and bone union rate when autologous bone graft techniques were applied. Therefore, bone defect size should not be the only factor used when choosing between vascularized or non vascularized bone graft. Finally, pooled analysis stated that in the presence of infection, vascularized graft has a lower risk of post-surgery infection. Well-designed randomized, controlled trials are needed to raise the low level of evidence for those conclusions.

## Additional files

Additional file 1: Table modified Coleman methodology score for bone defect. (DOCX 74 kb)
Additional file 2: Table studies excluded after full text review. (DOCX 99 kb)
Additional file 3: Table removed cases from included studies and technical notes. Technical notes represent values related to the entire sample of the studies where it was not possible to individualize data of the included cases. (DOCX 52 kb)
Additional file 4: Table additional procedures to achieve healing. (DOCX 60 kb)

## Abbreviations

PMMA: Polymethylmethacrylate
