# Supplementary material for: Autologous bone graft in the treatment of post-traumatic bone defects: a systematic review and meta-analysis
Source: BMC Musculoskelet Disord. 2016 Nov 9;17:465. doi: 10.1186/s12891-016-1312-4 (PMC5103502; doi:10.1186/s12891-016-1312-4)
Supplement: Additional file 1: — Table modified Coleman methodology score for bone defect. (DOCX 74 kb) [file 12891_2016_1312_MOESM1_ESM.docx]

**Additional file 1, table.** Modified Coleman methodology score for bone defect.

| **TABLE.** Modified Coleman methodology score for bone defect | |
| --- | --- |
| Part A. Only one score to be given for each of the seven sections | Score |
| **1. Study size** - Number of bone defects |  |
| > 60 | 10 |
| 41–60 | 7 |
| 20–40 | 4 |
| **<** 20, not stated | 0 |
| **2. Mean follow-up** (months) |  |
| > 24 | 5 |
| 12 – 24 | 2 |
| < 12, not stated or unclear | 0 |
| **3. Number of different graft techniques used in treatment** |  |
| One type of graft only | 10 |
| More than one type of graft but more than **>** 90% of subjects undergoing the same graft type | 7 |
| Not stated, unclear or **<** 90% of subjects undergoing the same graft type | 0 |
| **4. Type of study** |  |
| Randomized controlled trial | 15 |
| Prospective cohort study | 10 |
| Retrospective cohort study / Case series | 0 |
| **5. Diagnostic certainty** (clear definition of the bone defects, location, type, measurement technique) |  |
| All 4 definitions | 5 |
| 2 or 3 definitions | 3 |
| Less than 2 | 0 |
| **6. Description of surgical procedure given** |  |
| Adequate (technique stated and necessary details of that type of procedure given) | 5 |
| Fair (technique only stated without elaboration) | 3 |
| Inadequate, not stated or unclear | 0 |
| **7. Follow-up loss** |  |
| Well described with more than 80% of patients complying | 10 |
| Well described with less than 80% of patients complying | 5 |
| not reported | 0 |
| Part B. Scores may be given for each option in each of the three sections if applicable |  |
| **1. Outcome criteria** (if outcome criteria are vague and do not specify union rate and if was obtained with additional procedures score is automatically 0 for this section) |  |
| Outcome measures clearly defined | 2 |
| Timing of outcome assessment clearly stated (e.g., at best outcome after surgery or at follow-up) | 2 |
| Primary outcomes results clearly presented | 3 |
| Secondary outcome results clearly presented | 3 |
| **2. Procedure for assessing outcomes** |  |
| Subjects recruited (results not taken from surgeons’ files) | 5 |
| Investigator independent of surgeon | 4 |
| Written assessment | 3 |
| Completion of assessment by subjects themselves with minimal investigator assistance | 3 |
| **3. Description of subject selection process** |  |
| Selection criteria reported and unbiased | 5 |
| Recruitment rate reported > 80% | 5 |
| Recruitment rate reported < 80% | 3 |
| Eligible subjects not included in the study satisfactorily accounted for or 100% recruitment | 5 |
