# Supplementary material for: Autologous bone graft in the treatment of post-traumatic bone defects: a systematic review and meta-analysis
Source: BMC Musculoskelet Disord. 2016 Nov 9;17:465. doi: 10.1186/s12891-016-1312-4 (PMC5103502; doi:10.1186/s12891-016-1312-4)
Supplement: Additional file 2: — Table studies excluded after full text review. (DOCX 99 kb) [file 12891_2016_1312_MOESM2_ESM.docx]

**Additional file 2, table.** Studies excluded after full text review.

| 1. Guo QF, Xu ZH, Wen SF, et al. Value of a skin island flap as a postoperative predictor of vascularized fibula graft viability in extensive diaphyseal bone defect reconstruction. Orthop Traumatol Surg Res. 2012;98(5):576-82. |
| --- |
| 2. Fitoussi F, Masquelet AC, Rigal S, et al. Inter-tibiofibular graft for traumatic segmental bone defect of the tibia. Orthop Traumatol Surg Res. 2012;98(2):214-9. |
| 3. Karger C, Kishi T, Schneider L, et al. Treatment of posttraumatic bone defects by the induced membrane technique. Orthop Traumatol Surg Res. 2012;98(1):97-102. |
| 4. Donegan DJ, Scolaro J, Matuszewski PE, et al. Staged bone grafting following placement of an antibiotic spacer block for the management of segmental long bone defects. Orthopedics. 2011;34(11):e730-5. |
| 5. Zhen P, Liu XY, Lu H, et al. Fixation and reconstruction of severe tibial shaft fractures with vascularized fibular grafting. Arch Orthop Trauma Surg. 2011;131(1):93-9. |
| 6. Stafford PR, Norris BL. Reamer-irrigator-aspirator bone graft and bi Masquelet technique for segmental bone defect nonunions: a review of 25 cases. Injury. 2010;41(2):S72-7. |
| 7. Sun Y, Zhang C, Jin D, et al. Treatment for large skeletal defects by free vascularized fibular graft combined with locking plate. Arch Orthop Trauma Surg. 2010;130(4):473-9. |
| 8. Henry SL, Frome BA, Pederson WC. Vascularized bone transfer for severe injury around the ankle. Microsurgery. 2009;29(5):353-60. |
| 9. Oh JK, Bae JH, Oh CW, et al. Treatment of femoral and tibial diaphyseal nonunions using reamed intramedullary nailing without bone graft. Injury. 2008;39(8):952-9. |
| 10. Adani R, Delcroix L, Innocenti M, et al. Free fibula flap for humerus segmental reconstruction: report on 13 cases. Chir Organi Mov. 2008;91(1):21-6. |
| 11. Ristiniemi J. External fixation of tibial pilon fractures and fracture healing. Acta Orthop Suppl. 2007;78(326):3, 5-34. |
| 12. Kremer T, Bickert B, Germann G, et al. Outcome assessment after reconstruction of complex defects of the forearm and hand with osteocutaneous free flaps. Plast Reconstr Surg. 2006;118(2):443-54. |
| 13. Maneerit J, Meknavin S, Hanpanitkitkan S. Percutaneous versus open bone grafting in the treatment of tibial fractures: a randomized prospective trial. J Med Assoc Thai. 2004;87(9):1034-40. |
| 14. Muramatsu K, Ihara K, Shigetomi M, et al. Femoral reconstruction by single, folded or double free vascularised fibular grafts. Br J Plast Surg. 2004;57(6):550-5. |
| 15. Chen MT, Chang MC, Chen CM, et al. Double-strut free vascular fibular grafting for reconstruction of the lower extremities. Injury. 2003;34(10):763-9. |
| 16. Masquelet AC. Muscle reconstruction in reconstructive surgery: soft tissue repair and long bone reconstruction. Langenbecks Arch Surg. 2003;388(5):344-6. |
| 17. Falder S, Sinclair JS, Rogers CA, et al. Long-term behaviour of the free vascularised fibula following reconstruction of large bony defects. Br J Plast Surg. 2003;56(6):571-84. |
| 18. Borrelli J Jr, Prickett WD, Ricci WM. Treatment of nonunions and osseous defects with bone graft and calcium sulfate. Clin Orthop Relat Res. 2003;(411):245-54. |
| 19. Huang WC, Chen HC, Wei FC, et al. Chimeric flap in clinical use. Clin Plast Surg. 2003;30(3):457-67. |
| 20. Arai K, Toh S, Tsubo K, et al. Complications of vascularized fibula graft for reconstruction of long bones. Plast Reconstr Surg. 2002;109(7):2301-6. |
| 21. Yokoyama K, Itoman M, Nakamura K, et al. Free vascularized fibular graft vs. Ilizarov method for post-traumatic tibial bone defect. J Reconstr Microsurg. 2001;17(1):17-25. |
| 22. Minami A, Kasashima T, Iwasaki N, et al. Vascularised fibular grafts. An experience of 102 patients. J Bone Joint Surg Br. 2000;82(7):1022-5. |
| 23. Lee KS, Park JW. Free vascularized osteocutaneous fibular graft to the tibia. Microsurgery. 1999;19(3):141-7. |
| 24. Lin CH, Wei FC, Chen HC, et al. Outcome comparison in traumatic lower-extremity reconstruction by using various composite vascularized bone transplantation. Plast Reconstr Surg. 1999;104(4):984-92. |
| 25. Musharafieh R, Osmani O, Musharafieh U, et al. Efficacy of microsurgical free-tissue transfer in chronic osteomyelitis of the leg and foot: review of 22 cases. J Reconstr Microsurg. 1999;15(4):239-44. |
