# Supplementary material for: Autologous bone graft in the treatment of post-traumatic bone defects: a systematic review and meta-analysis
Source: BMC Musculoskelet Disord. 2016 Nov 9;17:465. doi: 10.1186/s12891-016-1312-4 (PMC5103502; doi:10.1186/s12891-016-1312-4)
Supplement: Additional file 3: — Table removed cases from included studies and technical notes. Technical notes represent values related to the entire sample of the studies where it was not possible to individualize data of the included cases. (DOCX 52 kb) [file 12891_2016_1312_MOESM3_ESM.docx]

**Additional file 3, table.** Removed cases from included studies and technical notes. Technical notes represent values related to the entire sample of the studies where it was not possible to individualize data of the included cases.

| **Authors** | **Excluded cases** | **Reason** | **Technical note** |
| --- | --- | --- | --- |
| Heitemman et al [19] | 3 | Bone defect secondary to tumor resection. | FU: 24 [mts] |
| Muramatsu et al [20] | 10 | Nonunion without bone defect | FU: 24 [mts] |
| Pelissier et al [15] | 7 | Amputation before bone defect treatment | Age: 34 [18 - 67]  Gender (m/f): 38/12 |
|  | 3 | In the sub-group analysis only 3 patients were enrolled in a induced membrane technique group | BD location (tibia/others): 39/4  FU: 84 [14 - 180] |
| Adani et al [30] | 1 | Bone defect secondary to treatment of Madelung’s deformity | FU: nr [10 - 93] |
| Jones et al [27] | 13 | Bone defects treated with recombinant human BMP-2 | FU: 11 cases returned  12 mts follow up visit |
| Allende et al [32] | 2 | Nonunion without bone defect (1)  Use of Allograft alone (1) | FU:19 [mts] |
| Ryzewicz et al [31] | 26 | Nonunion without bone defect | No |
| Niu et al [41] | 0 | No exclusion | BD size 1.9 [1 - 4]  Defect size in cm^3^ |

FU, follow up. m, male. f, female. nr, not reported. mts, months.
