# Supplementary material for: Autologous bone graft in the treatment of post-traumatic bone defects: a systematic review and meta-analysis
Source: BMC Musculoskelet Disord. 2016 Nov 9;17:465. doi: 10.1186/s12891-016-1312-4 (PMC5103502; doi:10.1186/s12891-016-1312-4)
Supplement: Additional file 4: — Table additional procedures to achieve healing. (DOCX 60 kb) [file 12891_2016_1312_MOESM4_ESM.docx]

**Additional file 4, table.** Additional procedures to achieve healing.

| **Authors** | **Healing problem** | | **Additional treatment** |
| --- | --- | --- | --- |
| Tu et al [17] | | 3 nonunions | bone graft (3 cases) |
| Toh et al [18] | | 1 delayed union | EF + bone graft (twice) |
| Heitmann et al [19] | | 3 nonunions | ORIF + bone graft (3 cases) |
| Muramatsu et al [20] | | 2 nonunions | ORIF + bone graft (2 cases) |
| Pelissier et al [15] | | 1 graft resorption | bone graft |
| Yajima et al [21] | | 1 nonunion | bone graft |
| Lee et al [22] | | 2 nonunions / 2 delayed unions | ORIF + bone graft (3 cases) / 1 nonunion infection with graft loss (failure) |
| Adani et al [23] | | 2 nonunions | bone graft (1 case) / no additional procedure (failure) (1 case) |
| Yazar et al [25] | | 5 nonunions | bone graft (2 cases) / not clear (3 cases) |
| Safoury [26] | | 1 nonunion | bone graft |
| Jones et al [27] | | 4 reinterventions | bone graft (2 cases) / implant fracture (2 cases) |
| Ristiniemi et al [29] | | 8 nonunions | bone graft (6 cases) (twice in one case) / exchange nailing (2 cases) |
| Adani et al [30] | | 3 nonunions | ORIF + bone graft (2 cases) / bone graft (1 case) |
| El-Gammal et al [48] | | nonunion | not reported |
| Ryzewicz et al [31] | | 1 nonunion | bone graft |
| Cavadas et al [33] | | 1 healing problem | bone graft |
| McCall et al [34] | | 8 nonunions | exchange nailing (2 cases) / ORIF + bone graft (1 case) / ORIF (1 case) / bone graft (1 case)  / infection debridement (2 cases) / recurrent nonunion (failure) 1 case |
| Sun et al [35] | | 1 nonunion | bone graft |
| Georgescu et al [39] | | 1 nonunion | fibula pedicle graft (1 case) (failure) |
| Liang et al [42] | | 1 nonunion | not clear |
| Niu et al [41] | | 1 delayed union | not clear |
| Özaksar et al [47] | | 3 nonunions | bone graft + EF (2 cases) / bone graft + cast (1 case) |

EF, external fixation. ORIF, open reduction and internal fixation. Failure, treatment failure.
